# Supplementary material for: A 1-week diet break improves muscle endurance during an intermittent dieting regime in adult athletes: A pre-specified secondary analysis of the ICECAP trial
Source: PLoS One. 2021 Feb 25;16(2):e0247292. doi: 10.1371/journal.pone.0247292 (PMC7906362; doi:10.1371/journal.pone.0247292)
Supplement: Attachment — Submitted filename: DietBreakStudy_Rebuttal.docx [file pone.0247292.s008.docx]

Dr. Cindy Gray
Academic Editor
*PLOS ONE*

plosone@plos.org

16th December

Re: Peos et al. manuscript [PONE-D-20-26641] - [EMID:336a102b9e7d99d2]

Dear Dr Gray,

Thank you for your email dated 10 November 2020 providing us with your and the Reviewer’s comments on our manuscript [PONE-D-20-26641] entitled *A 1-week diet break improves muscle endurance during an intermittent dieting regime in adult athletes*. We are pleased that our manuscript was noted to be well written and coherent. Please find attached our revised manuscript in which we have addressed all of the comments offered by you as the Editor as well as by the Reviewer, as outlined in our point-by-point rebuttal below. The changes have been marked in our revised manuscript. Please note, we have also uploaded the anonymized data sets necessary to replicate the study findings as Supporting Information files.

This trial was approved by the Human Research Ethics Committee at the University of Western Australia. Written informed consent was obtained from each eligible participant prior to inclusion. No data relating to individuals was identifiable in this trial.

This work was supported in part by Renaissance Periodisation and by the Australian Department of Education and Training via a Research Training Program Scholarship awarded to JJP, as well as by the National Health and Medical Research Council (NHMRC) of Australia via Senior Research Fellowships (1042555 and 1135897) to AS. The funders provided support in the form of salaries for authors AS and JP but did not have any additional role in the study design, data collection and analysis, decision to publish, or preparation of the manuscript. The specific roles of these authors are articulated in the ‘author contributions’ section.

AS reported owning 50% of the shares in Zuman International, which receives royalties for books she has written and payments for presentations at industry conferences; receiving presentation fees and travel reimbursements from Eli Lilly and Co, the Pharmacy Guild of Australia, Novo Nordisk, the Dietitians Association of Australia, Shoalhaven Family Medical Centres, the Pharmaceutical Society of Australia, and Metagenics; and serving on the Nestlé Health Science Optifast VLCD advisory board from 2016 to 2018. JK is employed by Weightology LLC. These affiliations do not alter our adherence to PLOS ONE policies on sharing data and materials.

We trust that the following satisfactorily addresses your and the Reviewer’s comments, and that our revised manuscript is now suitable for publication in *PLOS ONE.*

Yours sincerely,

Jackson Peos, PhD (c)

**Detailed responses to the Reviewer’s and Editor’s comments**

**Editor comments**

*1.	In general, the manuscript is coherent and well written with very few typos. However, there are concerns about the extent to which we can rely on these findings given the small sample size. The authors need to show that the study is powered to detect the changes (or lack of) described. If it is not, then it needs to be described as a pilot study, and the results discussed accordingly.*

**Our response:** Thank you for these comments. This study is a pre-specified secondary analysis of the ICECAP randomised controlled trial (RCT). As with RCTs, power calculations were performed only for the primary (i.e., co-primary) outcomes, and we did not perform power calculations for the secondary outcomes. With this in mind, all of the secondary outcomes of the ICECAP RCT – which is all of those in the current paper – are indeed exploratory variables. We acknowledge that we did not make this clear in the original manuscript title, and we have amended the title accordingly, as shown in marked text. Furthermore, as a priori power analyses were not conducted for this secondary exploratory study, we have modified the tone when discussing the results, shown in marked text in the Abstract and the Discussion section, notably in the second-to-last paragraph of the Discussion.

*2.	Abstract: line 26 to 28, should age not be used to describe the participants rather than “before the 1-week diet break”? Similarly, is height required to be reported here? Lines 41 to 44, the summary is confusing.*

**Our response:** Thank you for these comments, we can now appreciate how lines 26 to 28 in the Abstract of our original paper were confusing. We have amended these lines to provide more clarity, as shown in marked text in the top third of the Abstract. Height was originally included to provide additional description of the cohort; however, we agree it is not essential in the abstract, and subsequently have removed this detail. Finally, we have modified the text after ‘In summary’ at the end of the Abstract in order to clarify it, and we trust that this is clearer now.

*3.	Introduction: Please could you describe your pilot study more clearly from line 62. The aim of the study doesn't appear to fully fill the gap, which to my reading had been set out as understanding physiological, performance, cognitive and psychological changes during the diet break, rather than at the end of it.*

**Our response:** We have added additional detail to the final paragraph of the Introduction, stating 1) that this study fills the research gap by investigating the immediate effects of a diet break, and 2) that we are concerned with changes in outcomes *during* the diet break (i.e., comparing immediately before and after the diet break) as opposed to assessing outcome measures only at the *end* of the diet break. Additionally, we have changed the terminology from *during* the diet break to *immediately before and after* the diet break to provide greater clarity.

*4.	Method: Could you give the gender split of the participants in this study from the outset? Results should not be provided in the method (table one). Furthermore, in table one titles, you can just say 95% confidence interval, you don't need “lower limit to upper limit”. Line 177 to 178, I don't think you need to refer to the protocol here, or at least the way you do it seems strange.*

**Our response:** We have included the detail of the gender split of the 30 participants from which the current sample was recruited, in the Materials and Methods section, under the subheading *Eligibility criteria for participants*. We have also removed Table 1 from the Materials and Methods section. Moreover, we have removed the mention of “lower limit to upper limit” text in Table 1. Finally, we have removed our referral to the published protocol paper in the sub-section of the Materials and methods section that is now titled *Body weight, height, fat mass, fat-free mass, and resting energy expenditure*, as suggested.

*5.	Results: Line 230, age should be provided as part of the description of the participants rather than as part of the baseline measures. Again, it is not clear that height is important – furthermore, its measurements is not described in the method.*

*Line 238 to 240 seems to be in the wrong place.*

*Given the low number of participants in the study, it is quite useful to have the graphs of individual participant outcomes, but it might be helpful to consider which of these should be in the main part of the manuscript and which should go into an appendix (there is a lot of information here)? Also, it would be good to refer to why individual outcomes are useful in the main body of the text, otherwise, perhaps Table 1 is enough?*

*In addition, given only 13 participants provided blood samples, is it worth reporting these data here (particularly given the apparent high variability in some of the measures)?*

**Our response:** We have rearranged the first paragraph of the Results section so that age is provided as part of the description of participants rather than as part of baseline measures. With respect to height, we argue that height is important. While height may not be particularly important for an athlete population, it is important for potential future analyses which might investigate Body Mass Index (BMI), with BMI being a standardised measure. We have now also described the method by which height was measured, under the sub-section of the Materials and methods section that is now titled *Body weight, height, fat mass, fat-free mass, and resting energy expenditure.*

As suggested, we have moved lines 238 to 240 from the end of the first paragraph of the Results section to earlier in this paragraph, which we agree improves the flow of text.

We believe that all of the figures in our manuscript are important and merit inclusion in the main manuscript to provide a visual display of the data. Unlike Table 1 alone, the graphical representation of individual outcomes displays the trends in outcome measures during the diet break, as well as the inter-individual variation, both of which are important. We have added a statement to the second paragraph of the Results section to explicitly explain this. Furthermore, we do not believe that any of the figures should go into an appendix, because all figures are relevant to the paper, and relegating some of them to supplementary appendixes would be less convenient for readers.

We also believe that the data from analysis of blood samples is important to include in the current paper, because it compliments data on the other secondary outcome measures, e.g., fat-free mass and resting energy expenditure. For example, despite the notable inter-individual variation and small sample size, the group average trend lines – albeit not statistically significant – in plasma concentrations of testosterone and IGF-1, and Free T3, reflect the changes in fat-free mass and resting energy expenditure occurring during the diet break, respectively (**Fig. 1**). Thus, this may suggest to future researchers that these trends might reach statistical significance with a larger sample, providing potentially important information surrounding the physiological mechanisms underpinning diet breaks. This may also be helpful for future authors conducting meta-analyses.

*6.	Discussion: It appears a bit contradictory to read in line 324 that the results may lack practical significance for athletes and then go on to discuss the practical significance of the results. I am not sure that you need to keep referring back to the introduction in the discussion. Given the small sample size(s) in this study, it is important not to overplay the results. For example, to what extent is the study powered to detect the various changes? Should the next step be to repeat the study using a larger sample and a controlled design?*

**Our response:** Thank you for these comments, we have removed the line from the first paragraph of our Discussion section, where we stated that the results may lack practical significance for athletes. We agree that this line was confusing. Additionally, we have removed the referral back to the Introduction section within the second paragraph of the Discussion section. We agree with the Editor that it is important to not overplay the current results. Thus, as shown in marked text the second last paragraph of the Discussion section, we have stated that given the absence of a comparator group and lack of power calculations for these specific outcomes, that the results should be interpreted with caution. Furthermore, we have provided recommendations for future research, including the employment of an appropriately-powered sample size and a controlled design (with appropriate comparator group) to allow conclusions to be made with greater conviction.

***Journal Requirements***

*1.	Please ensure that your manuscript meets PLOS ONE's style requirements, including those for file naming. The PLOS ONE style templates can be found at*

[*https://journals.plos.org/plosone/s/file?id=wjVg/PLOSOne_formatting_sample_main_body.pdf*](https://journals.plos.org/plosone/s/file?id=wjVg/PLOSOne_formatting_sample_main_body.pdf) *and*

[*https://journals.plos.org/plosone/s/file?id=ba62/PLOSOne_formatting_sample_title_authors_affiliations.pdf*](https://journals.plos.org/plosone/s/file?id=ba62/PLOSOne_formatting_sample_title_authors_affiliations.pdf)

**Our response:** We have revised the manuscript to ensure that it meets all of PLOS ONE’s style requirements.

*2.	Please include additional information regarding the survey or questionnaire used in the study and ensure that you have provided sufficient details that others could replicate the analyses. For instance, please provide additional information regarding the development and validation of the questionnaire, and if the questionnaire is not under a copyright more restrictive than CC-BY, please include a copy, in both the original language and English, as Supporting Information.*

**Our response:** The questionnaire was designed in line with the previously published framework describing the scientific procedures used to assess the psychological and behavioural expressions of appetite (Blundell et al., 2010, listing below). This report also includes guidelines for the valid assessment of satiety, and the extent to which a change in hunger can stand alone as a measure of appetite control. As stated by the authors (Blundell J and colleagues), the objective of their report was to produce a set of guidelines on good practice in carrying out appetite research. We have amended the manuscript – in the sub-section of the Materials and methods section entitled “*Appetite*” – to highlight this point and to cite the reference framework, to provide further information to the reader regarding the validity of the questions included in the survey. All 8 questions are listed in the text of the manuscript.

- Blundell J, de Graaf C, Hulshof T, Jebb S, Livingstone B, Lluch A, Mela D, Salah S, Schuring E, van der Knaap H, Westerterp M. Appetite control: methodological aspects of the evaluation of foods. Obes Rev. 2010 Mar;11(3):251-70. doi: 10.1111/j.1467-789X.2010.00714.x.

*3.	Thank you for including your ethics statement:  "This study has been approved by the Human Research Ethics Committee at the University of Western Australia (RA/4/20/4340).". Please provide additional details regarding participant consent. In the ethics statement in the Methods and online submission information, please ensure that you have specified (1) whether consent was informed and (2) what type you obtained (for instance, written or verbal, and if verbal, how it was documented and witnessed). If your study included minors, state whether you obtained consent from parents or guardians. If the need for consent was waived by the ethics committee, please include this information.*

*If you are reporting a retrospective study of medical records or archived samples, please ensure that you have discussed whether all data were fully anonymized before you accessed them and/or whether the IRB or ethics committee waived the requirement for informed consent. If patients provided informed written consent to have data from their medical records used in research, please include this information.*

*Once you have amended this/these statement(s) in the Methods section of the manuscript, please add the same text to the “Ethics Statement” field of the submission form (via “Edit Submission”).*

**Our response:** We have added text to the first paragraph of the Materials and methods section on ethics approval, including information about consent. This study did not include minors, and the need for consent was not waived by the Ethics committee. This was not a retrospective study and did not involve archived samples.

*4.	Thank you for stating the following in the Competing Interests section:*

*"AS reported owning 50% of the shares in Zuman International, which receives royalties for books she has written and payments for presentations at industry conferences; receiving presentation fees and travel reimbursements from Eli Lilly and Co, the Pharmacy Guild of Australia, Novo Nordisk, the Dietitians Association of Australia, Shoalhaven Family Medical Centres, the Pharmaceutical Society of Australia, and Metagenics; and serving on the Nestlé Health Science Optifast VLCD advisory board from 2016 to 2018." We note that one or more of the authors are employed by a commercial company: Weightology LLC.*

*4.1.	Please provide an amended Funding Statement declaring this commercial affiliation, as well as a statement regarding the Role of Funders in your study. If the funding organization did not play a role in the study design, data collection and analysis, decision to publish, or preparation of the manuscript and only provided financial support in the form of authors' salaries and/or research materials, please review your statements relating to the author contributions, and ensure you have specifically and accurately indicated the role(s) that these authors had in your study. You can update author roles in the Author Contributions section of the online submission form.*

*Please also include the following statement within your amended Funding Statement.*

*“The funder provided support in the form of salaries for authors [insert relevant initials], but did not have any additional role in the study design, data collection and analysis, decision to publish, or preparation of the manuscript. The specific roles of these authors are articulated in the ‘author contributions’ section.”*

*If your commercial affiliation did play a role in your study, please state and explain this role within your updated Funding Statement.*

**Our response:** We have amended the financial disclosures of the online submission statement to declare the commercial affiliation for James Krieger. We have also reviewed the statements related to authors’ contributions, and they are accurate. Finally, we have included the requested additional funding statement detailing the role of funders in the online submission form, and we have noted this in the cover to this rebuttal letter.

*4.2.	Please also provide an updated Competing Interests Statement declaring this commercial affiliation along with any other relevant declarations relating to employment, consultancy, patents, products in development, or marketed products, etc.*

*Within your Competing Interests Statement, please confirm that this commercial affiliation does not alter your adherence to all PLOS ONE policies on sharing data and materials by including the following statement: "This does not alter our adherence to PLOS ONE policies on sharing data and materials.” (as detailed online in our guide for authors* [*http://journals.plos.org/plosone/s/competing-interests*](about:blank) *. If this adherence statement is not accurate and there are restrictions on sharing of data and/or materials, please state these. Please note that we cannot proceed with consideration of your article until this information has been declared.*

*Please include both an updated Funding Statement and Competing Interests Statement in your cover letter. We will change the online submission form on your behalf.*

*Please know it is PLOS ONE policy for corresponding authors to declare, on behalf of all authors, all potential competing interests for the purposes of transparency. PLOS defines a competing interest as anything that interferes with, or could reasonably be perceived as interfering with, the full and objective presentation, peer review, editorial decision-making, or publication of research or non-research articles submitted to one of the journals. Competing interests can be financial or non-financial, professional, or personal. Competing interests can arise in relationship to an organization or another person. Please follow this link to our website for more details on competing interests:* [*http://journals.plos.org/plosone/s/competing-interests*](about:blank)

**Our response:** We have provided an updated Competing Interests Statement declaring all commercial affiliations, including the affiliation of James Krieger to Weightology LLC. Furthermore we have confirmed that no affiliations altered the adherence to PLOS ONE policies.

*5.	We note that you have indicated that data from this study are available upon request. PLOS only allows data to be available upon request if there are legal or ethical restrictions on sharing data publicly. For more information on unacceptable data access restrictions, please see* [*http://journals.plos.org/plosone/s/data-availability#loc-unacceptable-data-access-restrictions*](http://journals.plos.org/plosone/s/data-availability#loc-unacceptable-data-access-restrictions)*.*

*In your revised cover letter, please address the following prompts:*

*a) If there are ethical or legal restrictions on sharing a de-identified data set, please explain them in detail (e.g., data contain potentially sensitive information, data are owned by a third-party organization, etc.) and who has imposed them (e.g., an ethics committee). Please also provide contact information for a data access committee, ethics committee, or other institutional body to which data requests may be sent.*

*b) If there are no restrictions, please upload the minimal anonymized data set necessary to replicate your study findings as either Supporting Information files or to a stable, public repository and provide us with the relevant URLs, DOIs, or accession numbers. For a list of acceptable repositories, please see* [*http://journals.plos.org/plosone/s/data-availability#loc-recommended-repositories*](http://journals.plos.org/plosone/s/data-availability#loc-recommended-repositories)*.*

*We will update your Data Availability statement on your behalf to reflect the information you provide.*

**Our response:** We have included the data set as a Supporting Information File, as requested, and noted it in the cover to this rebuttal letter.


***Reviewer's Responses to Questions***

*1.	Is the manuscript technically sound, and do the data support the conclusions?

The manuscript must describe a technically sound piece of scientific research with data that supports the conclusions. Experiments must have been conducted rigorously, with appropriate controls, replication, and sample sizes. The conclusions must be drawn appropriately based on the data presented.*

*Reviewer #1: Yes*

*2.	Has the statistical analysis been performed appropriately and rigorously?*

*Reviewer #1: Yes*

*3.	Have the authors made all data underlying the findings in their manuscript fully available?

The* [*PLOS Data policy*](http://www.plosone.org/static/policies.action#sharing) *requires authors to make all data underlying the findings described in their manuscript fully available without restriction, with rare exception (please refer to the Data Availability Statement in the manuscript PDF file). The data should be provided as part of the manuscript or its supporting information or deposited to a public repository. For example, in addition to summary statistics, the data points behind means, medians and variance measures should be available. If there are restrictions on publicly sharing data—e.g., participant privacy or use of data from a third party—those must be specified.*

*Reviewer #1: Yes*

*4.	Is the manuscript presented in an intelligible fashion and written in standard English?

PLOS ONE does not copyedit accepted manuscripts, so the language in submitted articles must be clear, correct, and unambiguous. Any typographical or grammatical errors should be corrected at revision, so please note any specific errors here.*

*Reviewer #1: Yes*

*5.	Review Comments to the Author

Please use the space provided to explain your answers to the questions above. You may also include additional comments for the author, including concerns about dual publication, research ethics, or publication ethics. (Please upload your review as an attachment if it exceeds 20,000 characters)*

*Reviewer #1: An observational study was conducted to determine the effects of a diet break on fat and fat-free mass, resting energy expenditure, muscle endurance, hunger sensations, and irritability. A total of 26 athletes were studied. No differences in fat mass were observed pre to post diet; however, a small increase in body weight was noted. Muscle endurance of the legs improved after the diet break; however, muscle strength did not differ. Significant differences were observed with respect to hunger sensations, prospective consumption, irritability and sensations of fullness.*

**Minor revisions:**

*1.	Line 218: Indicate the specific type of Wilcoxon test. The paired test, analogous to the paired t-test, is the Wilcoxon signed-rank test.*

**Our response:** We have amended this line to specify that we used the Wilcoxon signed-rank test, thank you.

*2.	Cite the statistical software used for the analysis.*

**Our response:** We have detailed the statistical software used for the analysis in the Materials and methods section, under the subheading *Statistical analysis.* Specifically, the software used was JASP (Version 0.14).

*3.	State and justify the study’s target sample size with a pre-study statistical power calculation.*

**Our response:** Please see our response to the Editor’s comment number 1, above.

*6.	PLOS authors have the option to publish the peer review history of their article (*[*what does this mean?*](https://journals.plos.org/plosone/s/editorial-and-peer-review-process#loc-peer-review-history)*). If published, this will include your full peer review and any attached files. If you choose “no”, your identity will remain anonymous, but your review may still be made public.

Do you want your identity to be public for this peer review? For information about this choice, including consent withdrawal, please see our* [*Privacy Policy*](https://www.plos.org/privacy-policy)*.*

*Reviewer #1: No*
